# Psychometric performance of the CAMPHOR and SF-36 in pulmonary hypertension

**DOI:** 10.1186/1471-2466-13-45

**Published:** 2013-07-12

**Authors:** James Twiss, Stephen McKenna, Louise Ganderton, Sue Jenkins, Mitra Ben-L’amri, Kevin Gain, Robin Fowler, Eli Gabbay

**Affiliations:** 1 Galen Research Ltd, Manchester, United Kingdom; 2 Royal Perth Hospital, Perth, Australia; 3 Lung Institute of Western Australia, Centre for Asthma, Allergy and Respiratory Research, University of Western Australia, Crawley, Australia; 4 School of Physiotherapy and Curtin Health Innovation Research Institute, Curtin University, Perth, Australia; 5 Discipline of Physiotherapy, Faculty of Health Sciences, The University of Sydney, Darlington, Australia; 6 Sir Charles Gairdner Hospital, Perth, Australia; 7 School of Medicine and Pharmacology, University of Western Australia, Perth, Australia; 8 School of Medicine, University of Notre Dame, Fremantle, Australia

## Abstract

**Background:**

The Cambridge Pulmonary Hypertension Outcome Review (CAMPHOR) and the Medical Outcomes Study Short Form 36 (SF-36) are widely used to assess patient-reported outcome in individuals with pulmonary hypertension (PH). The aim of the study was to compare the psychometric properties of the two measures.

**Methods:**

Participants were recruited from specialist PH centres in Australia and New Zealand. Participants completed the CAMPHOR and SF-36 at two time points two weeks apart. The SF-36 is a generic health status questionnaire consisting of 36 items split into 8 sections. The CAMPHOR is a PH-specific measure consisting of 3 scales; symptoms, activity limitations and needs-based QoL. The questionnaires were assessed for distributional properties (floor and ceiling effects), internal consistency (Cronbach's alpha), test-retest reliability and construct validity (scores by World Health Organisation functional classification).

**Results:**

The sample comprised 65 participants (mean (SD) age = 57.2 (14.5) years; n(%) male = 14 (21.5%)). Most of the patients were in WHO class 2 (27.7%) and 3 (61.5%). High ceiling effects were observed for the SF-36 bodily pain, social functioning and role emotional domains. Test-retest reliability was poor for six of the eight SF-36 domains, indicating high levels of random measurement error. Three of the SF-36 domains did not distinguish between WHO classes. In contrast, all CAMPHOR scales exhibited good distributional properties, test retest reliability and distinguished between WHO functional classes.

**Conclusions:**

The CAMPHOR exhibited superior psychometric properties, compared with the SF-36, in the assessment of PH patient-reported outcome.

## Background

Pulmonary hypertension (PH) is associated with progressive elevation of pulmonary artery pressure (PAP) and pulmonary vascular resistance (PVR), leading to right ventricular failure and premature death [1]. Pulmonary arterial hypertension is a rare condition with an estimated incidence of 2-7 per million per year [2,3]. However, incidence rates are considerably higher when other subtypes of PH are considered [4]. Previous research has indicated a higher prevalence in females of around 1.5 to 3 times that of men [3]. PH presents with nonspecific symptoms, including dyspnea on exertion, fatigue and syncope. These symptoms are often difficult to separate from those caused by other disorders, leading to late diagnosis [5]. Patients can experience severe limitations in physical activity requiring lifestyle modifications [6] and the inability to maintain employment [7]. The psychological impact of PH can result in social isolation, depression [8-10] and diminished quality of life [11].

Several types of outcome measure are available for determining the impact of PH. Haemodynamic variables, such as PVR, are often used as primary endpoints in clinical trials. However, evidence shows that these do not correlate well with the impact of the illness from the patients’ perspective [12]. Measures of physical function, such as the 6-minute walk distance (6MWD), are also frequently used. Although these measures provide objective data they do not capture the impact of the disease on patients. Researchers often use patient-reported outcome measures (PROMs) to determine the wider impact of PH from the patient’s perspective.

There are two main types of PROMs; generic and disease-specific. Generic outcome measures are used with a wide range of illnesses. These measures are popular as they are thought to negate the need to develop a new measure for each disease studied. One limitation of generic measures is that they may not assess concerns that are unique to each illness and important to patients. Disease-specific measures are developed to assess the specific concerns of the patient group [13].

The two most widely used PROMs with PH patients are the Medical Outcomes Study Short-Form 36 general health survey (SF-36) [14] and the Cambridge Pulmonary Hypertension Outcome Review (CAMPHOR) [15]. The SF-36 is a generic health-related quality of life (HRQL) measure that has been used in several clinical trials for PH. Despite this, limited information is available regarding the psychometric properties of the SF-36 in a PH population. Previous research has shown that the SF-36 correlates with functional measures such as the 6MWD and New York Heart Association assessment of functional class [12]. In addition, there is some evidence that the SF-36 is responsive in the PH population [16]. However, findings have been inconsistent and only some of the SF-36 domains appear to be responsive [17-19]. In addition, the investigation of scores representing the minimal important difference (MID) of the SF-36 in this patient group has shown that some of the domains of the SF-36 have large MID values [20]. This implies that large changes in scores are required to indicate a real change in health status.

The CAMPHOR is a PH-specific measure and comprises three scales assessing impairments (symptoms), activity limitations (functioning) and quality of life (QoL). A further development of the measure led to a utility scale for use in economic evaluations [21]. The content for the measure was derived directly from patient interviews and embodies issues important to patients with PH. The CAMPHOR has been shown to have good construct validity and reproducibility [15]. All three scales have been shown to fit the Rasch model providing evidence of unidimensionality. In addition, there is evidence that the scales are responsive to change [22]. Although the psychometric properties of the CAMPHOR are promising, direct comparisons with other measures are lacking.

The aim of this study was to conduct a direct comparison of the psychometric properties of the CAMPHOR and the SF-36 in a single population of PH patients in order to determine the suitability of each as an outcome measure.

## Methods

### Participants

The study utilizes data collected in Australia and New Zealand [23]. Participants were men and women over the age of 18 years, who met World Health Organisation (WHO) [24] criteria for the diagnosis of PH. Participants were required to be native English speaking and were excluded if they were unable to complete the questionnaires due to cognitive impairment. Ethics committees at Royal Perth Hospital and Curtin University in Australia gave approval for the study. Informed consent was obtained from the participants.

### Outcome measures

#### CAMPHOR

The CAMPHOR was developed in the United Kingdom (UK) [15] and subsequently adapted for use in Australia and New Zealand [23]. It consists of three scales; the Symptom Scale and QoL Scale both consist of 25 items with a dichotomous response format (Yes/No). Scores can range from 0-25 with a low score indicating minimal symptoms or better QoL. The Activity Scale consists of 15 items with a 3 point rating system (Able to do on own without difficulty/Able to do on own with difficulty/Unable to do on own). Scores range from 0-30 with a low score indicating minimal activity limitation.

#### SF-36; version 2

The SF-36 [14] is a generic health status questionnaire consisting of eight domains; physical functioning (10 items), social functioning (2 items), role limitations due to physical problems (4 items), role limitations due to emotional problems (3 items), mental health (5 items), energy/vitality (4 items), pain (2 items), general health perception (5 items) and a single health transition item. Raw domain scores are transformed to a scale of 0-100 with high scores indicating better health status.

### Procedure

Details of the methodology are reported in full elsewhere [23]. In brief, the study was conducted via postal survey. Participants completed the SF-36 and CAMPHOR at two time-points, two weeks apart. They also provided demographic and disease information (age, gender, WHO class and PH type). Participants completed the SF-36 immediately followed by the CAMPHOR at each time point (Time 1 [T1] and Time 2 [T2]).

### Statistical analyses

Data were analysed using SPSS Version 16.0. Data are provided for T1 and T2 assessment points throughout the results section.

### Distributional properties

The distributional properties of the CAMPHOR and SF-36 were examined using descriptive statistics including mean, standard deviation, median, inter-quartile range and range. The proportion of participants scoring the minimum and maximum possible scores on the questionnaires was also assessed. This provides an indication of the targeting of the questionnaire to the patient group. A high proportion of participants scoring at the extremes can indicate lack of sensitivity and/or relevance.

### Internal consistency

Internal consistency was assessed using Cronbach’s alpha coefficients for CAMPHOR and SF-36. This coefficient measures the extent to which items in a scale are inter-related. A low alpha (below 0.7) indicates insufficient relations between the items to form a scale [25].

### Test-retest reliability

The test-retest reliability of a measure is an estimate of its reproducibility over time when no change in the condition being assessed has taken place. The test-retest reliability of the CAMPHOR and the SF-36 was examined by correlating scores collected at T1 and T2 using Spearman’s rank correlation coefficients. A correlation coefficient greater than or equal to 0.85 is required to indicate that a scale has low random measurement error [26]. It is important to note that the Spearman’s correlation coefficient does not represent the percentage of explained variance. To assist with the interpretation of the correlation coefficient, the percentage of variance explained in the CAMPHOR and SF-36 scores (*r*^2^) was calculated. In addition, corresponding confidence intervals for mean scores were provided based on the standard error of measurement (SEM) to indicate the level of accuracy inherent in the scores. The SEM is useful for estimating how participants may score during repeated applications of the same measure. Confidence intervals based on the SEM show how participants’ scores are distributed around their ‘true scores’. Measures with lower reliability will have higher SEM values and wider confidence intervals. The SEM is defined in terms of the standard deviation (δ) and the reliability (r) as follows:

$$\mathrm{SEM}=\delta \surd \left(1-r\right)$$

### Construct validity (Known group validity)

Construct validity was determined using non-parametric tests for independent samples (Mann-Whitney U Test) to test for differences in CAMPHOR and SF-36 scores between groups according to disease severity (WHO functional classification). A *p* value of <0.05 was considered statistically significant.

## Results

### Descriptive statistics

Sixty-five participants (51 females, 78.5%) were recruited to the study. Demographic information for the sample is shown in Table 1.

**Table 1:** Demographics of the study subjects (n=65)

**Gender**	
Male (%)	14 (21.5)
Female (%)	51 (78.5)
**Age**	
Mean (SD)	57.2 (14.5)
Median (IQR)	57.8 (47.5-67.8)
Range	20.1-87.5
**WHO Classification**	
I (%)	3 (4.6)
II (%)	18 (27.7)
III (%)	40 (61.5)
IV (%)	4 (6.2)
**PH Type**	
Idiopathic PAH (%)	37 (56.9)
Familial PAH (%)	1 (1.5)
Associated PAH (%)	23 (35.4)
Chronic thromboembolic PH (%)	2 (3.1)
PH associated with lung diseases (%)	2 (3.1)

### Distributional properties

Total score descriptive information for the SF-36 is shown in Table 2. Results indicated that there were high levels of ceiling effects (% scoring maximum) for the bodily pain, social functioning and role-emotional domains of the SF-36 at both T1 and T2.

**Table 2:** Descriptive statistics for SF-36 domains

***Time 1***	**Physical functioning**	**Role-physical**	**Bodily pain**	**General health**	**Vitality**	**Social functioning**	**Role-emotional**	**Mental health**
	**59**	**60**	**61**	**60**	**61**	**61**	**60**	**61**
***n***								
**Median (IQR)**	35.0 (20.0-50.0)	37.5 (20.3-67.2)	52.0 (41.0-74.0)	30.0 (15.0-47.0)	37.5 (18.8-59.4)	75.0 (37.5-87.5)	75.0 (50.0-97.9)	65.0 (52.5-85.0)
**Mean (SD)**	35.3 (21.9)	41.9 (27.9)	53.4 (25.1)	30.3 (19.8)	38.2 (23.8)	62.1 (31.1)	67.9 (31.2)	67.4 (17.9)
**Range**	0.0-80.0	0.0-100.0	0.0-100.0	0.0-72.0	0.0-81.3	0.0-100.0	0.0-100.0	30.0-100.0
**Floor effect (% scoring min)**	5.1	5.0	4.9	8.3	6.6	3.3	6.7	3.3
**Ceiling effect (% scoring max)**	3.4	3.3	9.8	3.3	4.9	21.3	25.0	1.6
***Time 2***								
***n***	**59**	**60**	**60**	**58**	**61**	**60**	**61**	**61**
**Median (IQR)**	30.0 (20.0-50.0)	40.6 (25.0-73.4)	51.5 (33.5-74.0)	25.0 (13.8-42.8)	31.3 (18.8-53.1)	62.5 (37.5-87.5)	75.0 (50.0-95.8)	70.0 (55.0-85.0)
**Mean (SD)**	35.2 (21.7)	42.6 (28.0)	54.5 (26.5)	30.3 (21.3)	37.1 (21.3)	60.8 (29.4)	68.0 (28.3)	70.0 (18.4)
**Range**	0.0-90.0	0.0-100.0	0.0-100.0	0.0-87.0	0.0-81.3	0.0-100.0	0.0-100.0	25.0-100.0
**Floor effect (% scoring min)**	6.8	6.7	3.3	3.4	4.9	3.3	3.3	1.6
**Ceiling effect (% scoring max)**	1.7	1.7	13.3	1.7	1.6	20.0	24.6	3.3

Total scale score descriptive information for the CAMPHOR is shown in Table 3. Minimal levels of floor and ceiling effects were found at each time point indicating the scales were well matched to the disease severity levels of the participants.

**Table 3:** Descriptive statistics for CAMPHOR scales

***Time 1***	**Symptoms**	**Activities**	**QoL**
***n***	**65**	**65**	**65**
**Median (IQR)**	14.0 (7.0 – 18.5)	9.0 (5.0 – 14.5)	11.0 (4.0 – 16.0)
**Mean (SD)**	13.0 (6.0)	9.9 (5.9)	10.4 (6.5)
**Range**	2.0 – 23.0	0.0 – 24.0	0.0 – 23.0
**Floor effect (% scoring min)**	0.0	3.1	6.2
**Ceiling effect (% scoring max)**	0.0	0.0	0.0
***Time 2***			
***n***	**65**	**65**	**65**
**Median (IQR)**	11.0 (7.0 – 17.0)	10.0 (6.0 – 15.0)	12.0 (5.0 – 16.0)
**Mean (SD)**	12.5 (6.0)	10.8 (6.1)	10.8 (6.3)
**Range**	1.0 – 25.0	0.0 – 23.0	0.0 – 23.0
**Floor effect (% scoring min)**	0.0	4.6	3.1
**Ceiling effect (% scoring max)**	1.5	0.0	0.0

### Internal consistency

The Cronbach’s alpha coefficients for the SF-36 and CAMPHOR are shown in Table 4. Values were acceptable (>0.70) for all scales for both measures. This indicates that items are sufficiently related to form scales.

**Table 4:** Cronbach’s alpha coefficients for the SF-36 and CAMPHOR

		**Time 1**	**Time 2**
**SF-36**	Physical functioning	.90	.90
	Role-physical	.94	.95
	Bodily pain	.92	.93
	General health	.74	.77
	Vitality	.88	.86
	Social functioning	.91	.85
	Role-emotional	.94	.91
	Mental health	.78	.81
**CAMPHOR**	Symptoms	.89	.89
	Activities	.91	.91
	QoL	.91	.91

### Test-retest reliability

Test-retest reliability, confidence intervals for mean scores and percentage of explained variance for the SF-36 and CAMPHOR are shown in Table 5. Test-retest reliability was good for the SF-36 physical functioning and general health domains. Test-retest correlations were below 0.85 for all other SF-36 domains. These SF-36 domains also had wide confidence intervals for mean scores (indicating score inaccuracy) and had low levels of explained variance (r^2^ < 0.70).

**Table 5:** Test-retest reliability and explained variance

		**Test-retest**	**% of explained variance (r**^**2**^**)**	**Time 1 mean**	**SEM**	**Corresponding confidence intervals**
**SF-36**	Physical Functioning	.93	86	35.3	5.8	29.5-41.1
	Role-Physical	.81	66	41.9	12.2	29.7-54.1
	Bodily Pain	.72	52	53.4	13.3	40.1-66.7
	General Health Perceptions	.94	88	30.3	4.9	25.5-35.1
	Vitality	.78	61	38.2	11.2	27.0-49.4
	Social Functioning	.76	58	62.1	15.2	46.9-77.3
	Role-Emotional	.70	49	67.9	17.1	50.8-85.0
	Mental Health	.75	56	67.4	9.0	58.5-76.4
**CAMPHOR**	Symptoms	.86	74	13.0	2.2	10.8-15.2
	Activities	.87	76	9.9	2.1	7.8-12.0
	QoL	.94	88	10.4	1.6	8.8-12.0

Test-retest coefficients were good for all CAMPHOR scales, indicating low levels of random measurement error. In addition, the confidence intervals were narrow and the scales had high levels of explained variance (Table 5).

### Construct validity - Known group validity

Known group validity results are shown in Table 6 and 7. Several of the SF-36 domains distinguished between participants based on their WHO functional classification. However, the bodily pain and mental health domains did not discriminate between groups at either time point (Table 6). The role-emotional domain discriminated between groups at T1 but not T2 (Table 6).

**Table 6:** Mean (SD) SF-36 scores by WHO functional classification

	**Physical functioning**	**Role-physical**	**Bodily pain**	**General health**	**Vitality**	**Social functioning**	**Role-emotional**	**Mental health**
	**Mean (SD)**	**Mean (SD)**	**Mean (SD)**	**Mean (SD)**	**Mean (SD)**	**Mean (SD)**	**Mean (SD)**	**Mean (SD)**
***Time 1 (n)***	**59**	**60**	**61**	**60**	**61**	**61**	**60**	**61**
**WHO Classification**								
**I and II**	49.5 (21.6)	61.3 (26.9)	60.1 (25.9)	38.9 (18.5)	48.2 (20.5)	75.0 (27.4)	82.9 (19.3)	72.1 (17.1)
**III and IV**	27.9 (18.4)	31.4 (22.5)	49.9 (24.3)	25.7 (19.1)	33.0 (24.0)	55.3 (31.1)	59.8 (33.5)	64.9 (18.0)
***p *value**	<.001	<.001	.360	.009	.015	.014	.010	.165
***Time 2 (n)***	**59**	**60**	**60**	**58**	**61**	**60**	**61**	**61**
**WHO Classification**								
**I and II**	48.5 (23.8)	59.4 (20.7)	61.7 (25.8)	38.3 (20.7)	47.9 (20.9)	72.6 (24.2)	76.2 (23.3)	73.8 (16.7)
**III and IV**	28.3 (17.2)	34.2 (27.6)	50.6 (26.4)	26.4 (20.8)	31.4 (19.4)	54.5 (30.3)	63.8 (30.0)	68.0 (19.1)
***p *value**	.001	.001	.158	.034	.005	.027	.123	.167

*p* value, Mann-Whitney U-tests.

**Table 7:** Mean (SD) CAMPHOR scores by WHO functional classification

***Time 1***	**n**	**Symptoms**	**Activities**	**QoL**
		**Mean (SD)**	**Mean (SD)**	**Mean (SD)**
**WHO classification**				
**I and II**	21	10.4 (5.3)	7.2 (5.7)	7.3 (6.1)
**III and IV**	44	14.3 (5.9)	11.2 (5.7)	11.9 (6.2)
***p value***		0.012	0.011	0.007
***Time 2***				
**WHO classification**				
**I and II**	21	10.1 (5.3)	7.7 (6.2)	7.8 (5.8)
**III and IV**	44	13.6 (6.0)	12.3 (5.5)	12.2 (6.1)
***p value***		0.031	0.003	0.006

*p* value, Mann-Whitney U-tests.

The CAMPHOR was able to discriminate between participants based on WHO functional classification groups (I&II and III&IV) at T1 and T2. Significantly higher scores were found for WHO groups III and IV (Table 7).

## Discussion

This study compared the psychometric properties of two widely used PROMs for patients with PH. The results of the study showed that the CAMPHOR had excellent psychometric properties while weaknesses were apparent in several of the SF-36 domains.

Participants were predominantly in WHO classes II and III indicating moderately severe disease. Despite this three of the eight SF-36 domains (social functioning, role emotional and bodily pain) had high ceiling effects suggesting the participants in this study had no health problems. It is clear these domains lack sensitivity for this patient group. This could be due to the scales containing too few items (2-3 items each). It is also possible that the content of the items is not relevant to this patient group.

Six of the eight SF-36 domains demonstrated inadequate test-retest reliability (r<0.85).Two additional statistics were included to assist with interpreting this finding; the percentage of explained variance and standard error of measurement. The SF-36 domains that did not meet acceptable levels of reliability explained only 49-66% of variance in scores. These domains also had high SEM values and wide confidence intervals. Taken together, this indicates that six of the eight SF-36 domains had high levels of random measurement error and inaccuracy. The low reliability of these SF-36 domains suggests that these are not acceptable as a measure intended for use in clinical trials and other types of research in individuals with PH, where the ability to measure changes over time is important. Only the SF-36 physical functioning and general health domains met the required criteria in this sample. In contrast, all of the CAMPHOR domains met the test-retest criteria and showed low levels of random measurement error. This indicates that, unlike the SF-36 outcome, a change in CAMPHOR score is more likely to represent a real change in clinical condition and/or QoL.

Several of the SF-36 domains were able to distinguish between WHO functional classification groups. However, the bodily pain and mental health domains did not distinguish between groups at either time point and the role-emotional domain did not distinguish between groups at Time 2. Although the social functioning scale distinguished between groups the differences in scores failed to reach the thresholds published for the MIDs for this patient group [20]. These findings raise further doubts about the suitability of these domains of the SF-36 for use with this patient group. Emotional symptoms are important features of PH. It is likely that the role-emotional section is not specific enough to PH to measure the construct adequately.

A recent study by Matura et al [27] in the US associated CAMPHOR and SF-36 scores with symptom clusters in PH patients. They found that severity of symptoms was related to outcomes on both measures. However, they did not explore the psychometric performance of the measures. It was interesting to note that scores on the psycho-social domains of the SF-36 (as in the present study) were remarkably high.

Other researchers have investigated the functioning of the SF-36 physical (PCS) and mental (MCS) component summaries in PH patients [28]. Chen et al reported low levels of end effects for the MCS and PCS scales. Considerable doubt has been raised about the validity of the statistical methodology employed in the calculation of these scales [29-36]. Both the PCS and MCS scores are calculated by using factor coefficients from all eight domains. The PCS includes positively weighted coefficients from the physical domains of the measure but also negatively weighted coefficients from the mental domains. This means that in order to obtain the highest PCS scores it is necessary to both have high scores on the physical domains and low scores on the mental domains. The same is true of the MCS. Such an approach to measurement leads to anomalies, including the creation of artificially low end effects. Therefore it was decided not to report PCS or MCS scores in the present study.

Based on the findings of this study only the SF-36 physical functioning and general health perceptions domains met adequate psychometric criteria for use in research in individuals with PH. The general health perceptions section of the SF-36 is concerned with perceptions of health and illness beliefs and the physical functioning scale with functional limitations. These outcomes measure only a limited aspect of patients’ experience with PH. The results of this study demonstrate that the CAMPHOR is a more complete tool to assess the impact of PH from the patients’ perspective, with good psychometric properties in all scales.

As the CAMPHOR is a disease-specific measure the content is highly relevant to PH patients. The low levels of floor and ceiling effects and high test-retest reliability show the measure is sensitive and has low levels of random measurement error. This in turn suggests the CAMPHOR will be responsive to change. A previous research study has provided evidence of the responsiveness of the CAMPHOR [22].

Limitations of the study are noted. A relatively small sample was available so the results should be interpreted with some caution (n=65). However, this is typical of studies in this orphan disease [16,37,38]. A high proportion of females were included in the sample (78.5%). This reflects the gender ratio prevalence in PH patients [3]. The study was not designed to compare responsiveness of the two measures. Despite this, psychometric analyses suggest that the CAMPHOR scales would be more responsive. Overall, the study has provided a good indication of the psychometric properties of the two measures.

## Conclusions

Only the SF-36 physical functioning and general health perceptions domains met adequate psychometric criteria for use in research on individuals with PH. In contrast, all three CAMPHOR scales met the criteria. The CAMPHOR has superior psychometric properties to the SF-36 in the assessment of PH patient-reported outcome.

## Competing interests

The present work was unfunded. JT, SPM and MB are employees of Galen Research (GR). GR developed and own the copyright of the CAMPHOR. The other authors have no conflict of interest.

## Authors’ contributions

JT and SPM were involved in the design of the study. LG, SJ, KG, RF and EG were involved in data acquisition and management. JT, SPM and MB conducted the psychometric evaluation. All authors contributed to the interpretation of the results. The manuscript was drafted by JT and SPM and all authors contributed to its critical review. The final manuscript was approved by all authors.

## Pre-publication history

The pre-publication history for this paper can be accessed here:

http://www.biomedcentral.com/1471-2466/13/45/prepub
